# Reduced sphingolipid biosynthesis modulates proteostasis networks to enhance longevity

**DOI:** 10.18632/aging.204485

**Published:** 2023-01-14

**Authors:** Nathaniel L. Hepowit, Eric Blalock, Sangderk Lee, Kimberly M. Bretland, Jason A. MacGurn, Robert C. Dickson

**Affiliations:** 1 Department of Cell and Developmental Biology, Vanderbilt University, Nashville, TN 37240, USA; 2 Department of Pharmacology and Nutritional Science, University of Kentucky, Lexington, KY 40536, USA; 3 College of Pharmacy, University of Kentucky, Lexington, KY 40536, USA; 4 Department of Molecular and Cellular Biochemistry, University of Kentucky, Lexington, KY 40536, USA

**Keywords:** longevity, ubiquitin, proteostasis, amino acid transport, sphingolipid

## Abstract

As the elderly population increases, chronic, age-associated diseases are challenging healthcare systems around the world. Nutrient limitation is well known to slow the aging process and improve health. Regrettably, practicing nutrient restriction to improve health is unachievable for most people. Alternatively, pharmacological strategies are being pursued including myriocin which increases lifespan in budding yeast. Myriocin impairs sphingolipid synthesis, resulting in lowered amino acid pools which promote entry into a quiescent, long-lived state. Here we present transcriptomic data during the first 6 hours of drug treatment that improves our mechanistic understanding of the cellular response to myriocin and reveals a new role for ubiquitin in longevity. Previously we found that the methionine transporter Mup1 traffics to the plasma membrane normally in myriocin-treated cells but is not active and undergoes endocytic clearance. We now show that *UBI4*, a gene encoding stressed-induced ubiquitin, is vital for myriocin-enhanced lifespan. Furthermore, we show that Mup1 fused to a deubiquitinase domain impairs myriocin-enhanced longevity. Broader effects of myriocin treatment on ubiquitination are indicated by our finding of a significant increase in K63-linked ubiquitin polymers following myriocin treatment. Although proteostasis is broadly accepted as a pillar of aging, our finding that ubiquitination of an amino acid transporter promotes longevity in myriocin-treated cells is novel. Addressing the role of ubiquitination/deubiquitination in longevity has the potential to reveal new strategies and targets for promoting healthy aging.

## INTRODUCTION

Aging is widely accepted as a major risk factor for many chronic diseases and resultant physiological decline leading to mortality [1]. Research on many fronts is revealing potential ways to postpone age-related decline, maintain normal physiological function longer and improve healthspan. Some of the most promising research seeks to limit nutrient intake or increase daily fasting time as a means to improve healthspan in humans [2–4]. Still, these strategies will be difficult for most humans to adhere to in order to gain health benefits. Pharmacological agents offer a potential way to obtain the beneficial effects of nutrient limitation, but such compounds have yet to be identified although progress is encouraging [1, 5, 6].

We have identified a potential pharmacological agent, the natural product myriocin (Myr, ISP-1), that increases chronological lifespan in budding yeasts (*Saccharomyces cerevisiae*) by more than two-fold [7]. Myr works, at least in part, by reducing the free pool of most amino acids similar to what amino acid restriction does [5, 8, 9]. Our interest in Myr stems from its target enzyme serine palmitoyltransferase (SPT), catalyzing the first and rate limiting step in sphingolipid biosynthesis in all eukaryotes [10–12]. In addition, Myr was first identified in a search for antibiotics [13] and anti-inflammatory drugs [14]. More recently, it has shown beneficial effects in treating age-associated diseases including diabetes, cancers, neurological and cardiovascular disorders [15–20] and other diseases including muscular dystrophies, cystic fibrosis and retinopathy [21–23].

Sphingolipids serve as both structural components of cellular membranes and as signal or regulatory molecules influencing many physiological processes, particularly in mammals [15, 24–26]. Because most de novo lipid biosynthesis begins in the endoplasmic reticulum and continues in the Golgi Apparatus before the terminal products are distributed to cellular membranes, Myr treatment has the potential to diminish or enhance a variety of processes. Our recent studies identified diminished processes that may foster longer lifespan: newly synthesized Mup1, the major high-affinity methionine (Met) transporter, trafficked normally to the plasma membrane (PM) but was inactive in drug-treated cells resulting in reduced Met uptake, starting after about 2 h of drug treatment, as the fraction of active Mup1 was diluted by cell growth and division. Moreover, Myr promoted endocytic clearance of Mup1, indicating that altered sphingolipid levels trigger remodeling of nutrient transporter composition at the PM [7]. Thus, post-translational effects are vital to Myr-induced down-sizing of amino acid pools.

Previously we found that Myr treatment had large, global effects on transcription after 6–7 cell doublings [27]. In the present work, we examined mRNA levels during the initial stages of Myr treatment to construct an overview of transcriptional changes with the aim of identifying how long it takes cells to respond to drug treatment and to identify novel factors critical for Myr-enhanced lifespan. We find that transcription is strongly up-regulated starting after 4 h of Myr treatment when cells progress through a second cell division cycle. In addition, transcript data suggested a novel role for ubiquitin in lifespan and targeted studies identified ubiquitination of Mup1 as essential for Myr-enhanced longevity. Our transcriptomics data provide a valuable resource to better understand how myriocin treatment enhances longevity.

## RESULTS

### Four hours of myriocin treatment induce robust transcriptional changes

**Figure 1:**
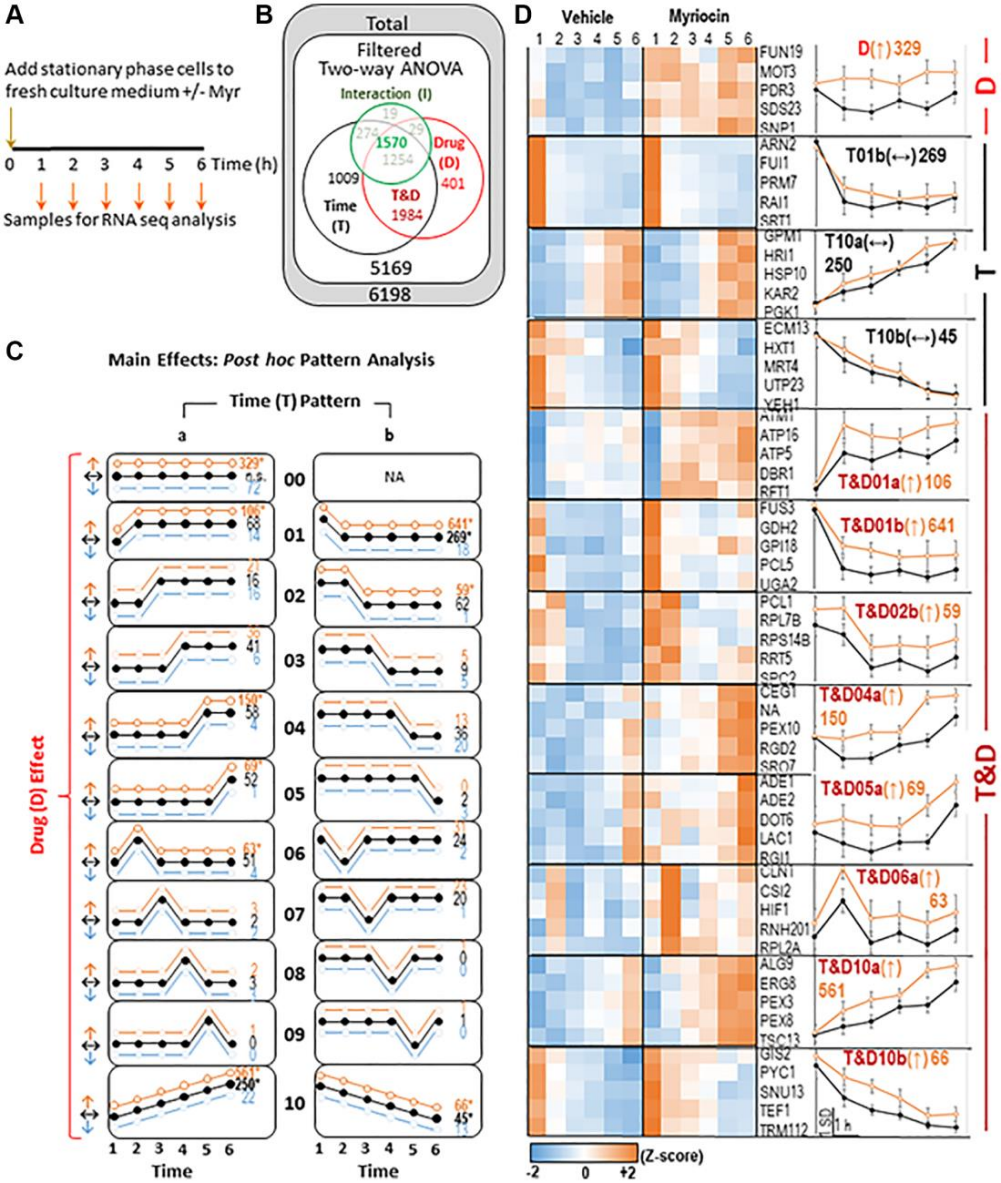
**Summary of transcriptomics data.** (**A**) Experimental Design- samples were taken every hour as indicated from vehicle (Veh) and myriocin (Myr) treated cultures. RNA was isolated and mRNA was quantified using RNA-seq. (**B**) Differentially expressed genes (DEGs)-RNA-seq data were filtered to include well-annotated genes with sufficient signal intensity prior to statistical analysis. Two-way ANOVA (Time: 6 time points; Drug: two conditions- Veh and Myr) was applied. The ANOVA test produces 3 *p*-values for each gene, one for the main effect of time (T- black circle), one for the main effect of drug (D- red circle), and one for the ‘Interaction’ between Time and Drug (I- green circle; analyzed separately- see Figure 2). DEGs (*p* ≤ 0.01 on at least one of the three *p*-values) are shown. Because each DEG could be significant by 1-3 ANOVA *p*-values, data are displayed in a Euler diagram to indicate overlap. (**C**) For DEGs significant by Time (T), Drug (D), or both (T&D), *post hoc* analysis categorized each DEG into one of 11 different temporal patterns (00- no temporal effect; 01 and 05- changed at the first or last time point; 02–04- plateaus at intermediate time points; 06–09- spikes at intermediate time points; 10- linear change with time; for each temporal pattern other than ’00’, a gene could be assigned to one of two horizontal reflections- ‘a’ or ‘b’), and within each temporal pattern, one of three drug effects (increased by Myr- ↑ orange, decreased by Myr- ↓ blue, not changed by Myr- ↔ black) was defined. Numbers of genes assigned to each pattern are included on the right side of each pattern diagram (^*^ significantly more DEGs than expected by chance; *p* ≤ 0.01, binomial test). (**D**) Results for each asterisked pattern from C are shown. On the left, heatmaps of standardized average signal at each time point for vehicle and myriocin groups for 5 representative DEGs are shown. On the right, the graphed standardized averages (± SD) for all genes assigned to each pattern are shown (scale bar at bottom). Pattern names are given by the Euler diagram region, then the temporal pattern # and ‘a’ or ‘b’ designation, followed by drug effect (in parentheses) and the number of genes assigned. All data for the 5169 RNA seq transcripts that passed filtering metrics are shown in Supplementary Table 1 along with the two-way ANOVA analysis results which can be used to sort for genes in each sector of the Euler diagram.

To examine transcriptional changes induced by Myr treatment, we diluted stationary phase prototrophic BY4741 cells (50–60% of cells synchronized for first two cell cycles over a 6 h time frame [7]) into fresh culture medium (Time 0), with and without Myr treatment. Samples for analysis of mRNA abundance by RNA seq were taken at 1 h intervals over a 6 h time course (Figure 1A). The normalized RNA seq data for the time course contained transcripts, expressed as transcripts per million (TPM), mapped to 6198 genes, 5169 of which were uniquely annotated and of sufficient signal intensity for subsequent analysis (Supplementary Table 1, Filter tab). Filtered data were analyzed by two-way ANOVA with a statistical cutoff of *p* = ≤ 0.01 to give a set of 4964 significant genes (Figure 1B). These were further sorted into drug (D), time (T), both time and drug (T&D) and Interactions (I) (Figure 1B, Euhler diagram), and assigned a *p*-value. Genes found to be significant in any of these three categories are referred to as differentially expressed (DEG).

**Figure 2:**
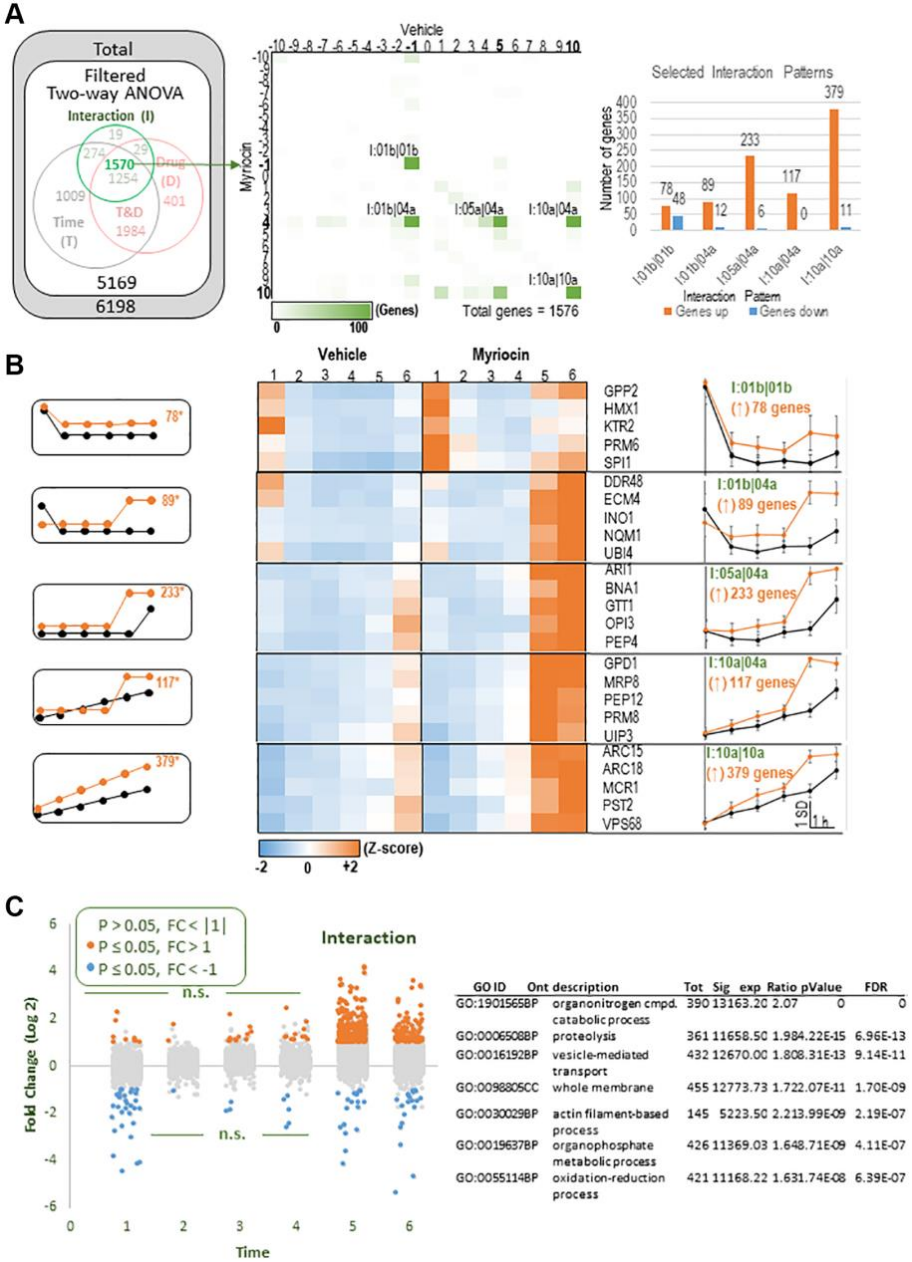
**Detailed analysis of the Interaction group.** (**A**) Left: Euler diagram of significant genes (also in Figure 1), focusing on the 1576 DEGs rated significant by the two-way ANOVA ‘interaction’ term. Center: A grid of all possible temporal patterns of expression for Vehicle samples (horizontal) and Myriocin samples (vertical) was used to count the number of Interaction DEGs assigned to each pattern. Results are shown as a heatmap with green highlighting. There are five patterns of interest (labels indicated) that each showed >100 DEGs (total results for this grid are available in Supplementary Table 1, tab called ‘I Intersections’). Right: The number of interaction-significant DEGs within each of these five patterns of interest are graphed according to whether Myr increased (orange) or decreased (blue) expression levels compared to vehicle-treated samples. Myr’s effect was predominantly to increase expression. (**B**) Left: Templates of expression for the five Myr-increased patterns of interest. Center: Heatmaps showing signal intensities for representative genes within each statistically identified pattern. Right: average Z-scored expression levels for all genes assigned to each selected pattern (functional overrepresentation analysis results for these patterns are presented in Table 2). (**C**) Left: The analysis of interaction-significant DEGs, particularly in 2B, suggested a simplified and more general trend among the genes found to be significant by the two-way ANOVA’s Interaction term. That is, Myr treatment appeared to increase expression at time points 5 and 6 regardless of the temporal pattern of expression. To test this, we plotted the log 2-fold changes of Myr treatment/vehicle treatment at each time point, and color-coded those results if they were significantly (*p* ≤ 0.05, pairwise Fisher’s LSD) downregulated (blue) or upregulated (orange). As expected, the predominant effect was upregulation at time points 5 and 6. Right: Functional overrepresentation analysis. Columns- Tot: total number of genes in the dataset assigned to GO term; Sig: number of total genes that were significant and were assigned to the indicated pattern of expression; Exp: number of genes expected to be found in that pathway for that pattern of expression by chance; Ratio: Sig/Exp; *p*-value: overrepresentation analysis ([46]); FDR: multiple testing adjusted ORA *p*-alue according the BH procedure ([47]; Note- complete list of all pathways, and the genes assigned to them, is provided in Supplementary Table 2, Webgestalt patterns combined tab).

DEGs that change up or down over time were identified by using a ‘template assignment tool’ (MATERIALS AND METHODS, example in Figure 2A, center panel). Most DEGs (99.86%) were assigned to a template. For DEGs significant by Time (T), Drug (D), or both (T&D), *post hoc* analysis categorized each DEG into one of 11 different temporal patterns plus one with no temporal effect (Figure 1C, Supplementary Table 1 - Pattern Graph tab can be used to plot the pattern of any gene). Within each temporal pattern (other than ’00’) a gene was placed in one of two horizontal reflections (Figure 1C, columns ‘a’ or ‘b’). Three types of Drug effects are shown in each pattern (Figure 1C, Y-axis: light blue, black or orange curves) along with the number of genes in each category (see Legend for Figure 1C for more details). These 11 patterns explained the majority (77%) of all DEGs. Results of DEGs in significantly enriched patterns are shown as a heatmap and a graph for Vehicle and Myriocin treatments (Figure 1D, see Legend for details and pattern labels). A graph of any gene transcript across the 1–6 h time-frame can be plotted using the Graph Reporter tab in Supplementary Table 1. The most significant GO terms enriched in these patterns are shown in Table 1 and a complete list of genes in each GO term is presented in Supplementary Table 2 (Webgestalt patterns combined tab). These GO terms primarily represent major processes, cellular compartments or molecular functions needed to drive cell growth and division such as chromatin organization, transcription regulation, mitotic cell cycle and biochemical pathways.

**Table 1:** Gene ontology overrepresentation analysis (Webgestalt) for the interaction group of the Euler diagram.

**Pattern**	**GO ID**	**Ont**	**Description**	**Tot**	**Sig**	**exp**	**Ratio**	***p*-value**	**FDR**
D(↑)	GO:0006325	BP	chromatin organization	343	50	22.26	2.25	2.12E-08	6.99E-06
	GO:0044427	CC	chromosomal part	470	56	30.50	1.84	3.04E-06	2.92E-04
	GO:0051172	BP	negative regulation of nitrogen compound metabolic process	436	53	28.30	1.87	3.20E-06	2.92E-04
	GO:0140110	MF	transcription regulator activity	261	37	16.94	2.18	3.54E-06	2.92E-04
	GO:0010629	BP	negative regulation of gene expression	431	52	27.97	1.86	5.04E-06	3.33E-04
	GO:0006357	BP	regulation of transcription by RNA polymerase II	453	53	29.40	1.80	1.01E-05	4.77E-04
T&D01a(↑)	GO:0000278	BP	mitotic cell cycle	388	29	8.24	3.52	7.68E-10	1.27E-07
	GO:0048285	BP	organelle fission	283	24	6.01	4.00	2.55E-09	2.80E-07
	GO:0007010	BP	cytoskeleton organization	273	21	5.79	3.62	1.67E-07	1.10E-05
	GO:0000003	BP	reproduction	474	28	10.06	2.78	2.94E-07	1.62E-05
	GO:0016817	MF	hydrolase activity, acting on acid anhydrides	424	25	9.00	2.78	1.57E-06	5.75E-05
	GO:0044427	CC	chromosomal part	470	26	9.98	2.61	3.10E-06	1.02E-04
T01b(↔)	GO:0022613	BP	ribonucleoprotein complex biogenesis	494	50	25.30	1.98	1.14E-06	3.04E-04
	GO:0005730	CC	nucleolus	283	34	14.50	2.35	1.84E-06	3.04E-04
	GO:0034660	BP	ncRNA metabolic process	493	45	25.25	1.78	6.37E-05	0.00701
	GO:0032259	BP	methylation	161	20	8.25	2.43	1.77E-04	0.014594
	GO:0140098	MF	catalytic activity, acting on RNA	245	25	12.55	1.99	6.42E-04	0.037068
	GO:0016071	BP	mRNA metabolic process	311	29	15.93	1.82	0.001047	0.043178
T&D01b(↑)	GO:0022613	BP	ribonucleoprotein complex biogenesis	494	221	62.91	3.51	<1E-16	<1E-16
	GO:0034660	BP	ncRNA metabolic process	493	215	62.78	3.42	<1E-16	<1E-16
	GO:0005654	CC	nucleoplasm	365	111	46.48	2.39	<1E-16	<1E-16
	GO:0005730	CC	nucleolus	283	161	36.04	4.47	<1E-16	<1E-16
	GO:0140098	MF	catalytic activity, acting on RNA	245	99	31.20	3.17	<1E-16	<1E-16
	GO:0032259	BP	methylation	161	68	20.50	3.32	<1E-16	<1E-16
T&D02b(↑): N.S.									
T&D04a(↑)	GO:0098798	CC	mitochondrial protein complex	204	17	6.12	2.78	1.08E-04	0.035628
	GO:0005759	CC	mitochondrial matrix	237	18	7.11	2.53	2.20E-04	0.036326
T&D05a(↑): N.S.									
T&D06a (↑)	GO:0044445	CC	cytosolic part	209	13	2.64	4.92	1.39E-06	4.59E-04
	GO:0005856	CC	cytoskeleton	255	12	3.23	3.72	6.39E-05	0.007031
	GO:0044427	CC	chromosomal part	470	16	5.95	2.69	1.68E-04	0.00958
T&D10a(↑)	GO:0016192	BP	vesicle-mediated transport	432	92	48.58	1.89	1.69E-10	2.80E-08
	GO:0098805	CC	whole membrane	455	91	51.16	1.78	6.79E-09	7.47E-07
	GO:0005794	CC	Golgi apparatus	282	64	31.71	2.02	1.12E-08	9.28E-07
	GO:0031982	CC	vesicle	279	62	31.37	1.98	4.54E-08	2.50E-06
	GO:0098796	CC	membrane protein complex	320	67	35.98	1.86	1.46E-07	6.88E-06
	GO:0061919	BP	process utilizing autophagic mechanism	194	42	21.82	1.93	1.53E-05	5.62E-04
	GO:0006886	BP	intracellular protein transport	472	81	53.08	1.53	3.35E-05	0.001106
T10a(↔)	GO:0005975	BP	carbohydrate metabolic process	271	35	13.49	2.59	1.07E-07	3.52E-05
	GO:0044432	CC	endoplasmic reticulum part	394	39	19.62	1.99	1.82E-05	0.003009
	GO:1901135	BP	carbohydrate derivative metabolic process	366	36	18.23	1.98	4.59E-05	0.005049
	GO:0042175	CC	nuclear outer membrane-ER membrane network	383	36	19.07	1.89	1.19E-04	0.009846
	GO:0005794	CC	Golgi apparatus	282	28	14.04	1.99	2.96E-04	0.016268
	GO:0006457	BP	protein folding	111	15	5.53	2.71	3.45E-04	0.016286
	GO:0098796	CC	membrane protein complex	320	30	15.93	1.88	4.85E-04	0.019998
	GO:0072524	BP	pyridine-containing compound metabolic process	94	13	4.68	2.78	6.84E-04	0.02507
	GO:0098805	CC	whole membrane	455	38	22.66	1.68	8.52E-04	0.026987
T&D10b (↑)	GO:0034660	BP	ncRNA metabolic process	493	24	5.94	4.04	5.05E-10	1.67E-07
	GO:0022613	BP	ribonucleoprotein complex biogenesis	494	23	5.95	3.87	3.30E-09	5.44E-07
	GO:0016741	MF	transferase activity, transferring one-carbon groups	100	9	1.20	7.47	2.31E-06	1.90E-04
	GO:0051169	BP	nuclear transport	179	9	2.16	4.18	2.46E-04	0.009008
T10b(↔): N.S.									

Columns- Ont: Abbreviations: BP: Biological Process; CC: Cellular Compartment; MF: Molecular function; Tot: total number of genes in the dataset assigned to GO term; Sig: number of total genes that were significant and were assigned to the indicated pattern of expression; Exp: number of genes expected to be found in that pathway for that pattern of expression by chance; Ratio: Sig/Exp. *p*-value: overrepresentation analysis (ORA- [46]); FDR: multiple testing adjusted ORA *p*-value according the BH procedure ([47]). Note- complete list of all pathways, and the genes assigned to them, is provided in Supplementary Table 2.

**Table 2:** Overrepresentation analysis of expression patterns for DEGs significant by the two-way ANOVA in the interaction group.

**Pattern**	**GO ID**	**Ont**	**Description**	**Tot**	**Sig**	**Exp**	**Ratio**	***p*-value**	**FDR**
I: 01b|01b(↑)	GO:0044283	BP	small molecule biosynthetic process	386	16	36.04	4.47	1.82E-04	0.036084
I: 01b|01b(↑)	GO:0006811	BP	ion transport	358	15	31.20	3.17	2.67E-04	0.036084
I: 01b|01b(↑)	GO:0055085	BP	transmembrane transport	425	16	20.50	3.32	5.45E-04	0.036084
I: 01b|01b(↑)	GO:0030312	CC	external encapsulating structure	124	8	5.94	4.04	5.47E-04	0.036084
I: 01b|01b(↑)	GO:0006082	BP	organic acid metabolic process	433	16	5.95	3.87	6.71E-04	0.03688
I:01b|04a N.S.									
I: 05a|04a(↑)	GO:0055114	BP	oxidation-reduction process	421	35	1.20	7.47	2.54E-04	0.02852
I: 05a|04a(↑)	GO:0005773	CC	vacuole	262	25	2.16	4.18	2.73E-04	0.02852
I: 05a|04a(↑)	GO:0051186	BP	cofactor metabolic process	266	25	5.91	2.71	3.46E-04	0.02852
I: 05a|04a(↑)	GO:0098805	CC	whole membrane	455	36	5.48	2.74	5.40E-04	0.033417
I: 05a|04a(↑)	GO:1901565	BP	organonitrogen compound catabolic process	390	32	6.51	2.46	6.08E-04	0.033417
I: 05a|04a(↑)	GO:0016788	MF	hydrolase activity, acting on ester bonds	279	25	1.90	4.22	7.10E-04	0.033493
I: 05a|04a(↑)	GO:0016311	BP	dephosphorylation	149	16	6.63	2.41	0.001035	0.042687
I: 10a|04a(↑)	GO:0016192	BP	vesicle-mediated transport	432	31	19.07	1.83	2.77E-09	9.13E-07
I: 10a|04a(↑)	GO:1901565	BP	organonitrogen compound catabolic process	390	20	11.87	2.11	3.89E-04	0.031287
I: 10a|10a(↑)	GO:0006508	BP	proteolysis	361	62	12.05	2.07	2.87E-10	4.74E-08
I: 10a|10a(↑)	GO:1901565	BP	organonitrogen compound catabolic process	390	64	20.61	1.75	1.02E-09	1.12E-07
I: 10a|10a(↑)	GO:0016192	BP	vesicle-mediated transport	432	68	17.67	1.81	1.77E-09	1.46E-07
I: 10a|10a(↑)	GO:0098805	CC	whole membrane	455	69	12.64	1.98	6.73E-09	4.44E-07
I: 10a|10a(↑)	GO:0098796	CC	membrane protein complex	320	53	6.75	2.37	2.53E-08	1.39E-06
I: 10a|10a(↑)	GO:0043248	BP	proteasome assembly	35	14	9.79	3.17	9.45E-08	3.90E-06
I: 10a|10a(↑)	GO:0030029	BP	actin filament-based process	145	30	8.83	2.26	2.87E-07	9.46E-06

Columns- Abbreviations: Tot: total number of genes in the dataset assigned to GO term; Sig: number of total genes that were significant and were assigned to the indicated pattern of expression; Exp: number of genes expected to be found in that pathway for that pattern of expression by chance; Ratio: Sig/Exp; *p*-value: overrepresentation analysis ([46]); FDR: multiple testing adjusted overrepresentation analysis *p*-value according the BH procedure ([47]). Note- complete list of all pathways, and the genes assigned to them, is provided in Supplementary Table 2.

In contrast to the Time x Drug sector, the 1009 genes in the Time sector of the Euler diagram fall primarily into three patterns (Figure 1C and 1D, T01b(↔), T10A (↔) and T10b(↔)). Genes in these patterns are not significantly changed by Myr treatment but are significantly changed with time and serve as an example of time-related changes. All of these patterns will require further effort to determine their significance, if any, in Myr-induced longevity.

The Drug sector of the Euler diagram (Figure 1C and 1D, pattern D(↑)) contains 329 up-regulated genes (Table 1 and Supplementary Table 2). Because of our experimental design, we cannot be sure if some genes in this sector are driving the changes associated with time that we see, but they could be. The most enriched GO terms relate to chromosomes, and transcription (Table 1), indicating a strong response to Myr treatment independent of time in culture.

Next, we analyzed the 1570 genes in the Interaction group for temporal patterns responding to Myr treatment (Figure 2A). Five patterns contained more than 100 DEGs (Figure 2A, center panel, numeric results for this grid are available in Supplementary Table 1, tab called ‘I intersections’). The right-hand graph in Figure 2A indicates the number of up- and down-regulated genes in these 5 patterns. Five templates for these patterns (Figure 2B, left-side graphs) along with heatmaps of representative genes in each pattern for Vehicle and Myriocin treated samples are shown in Figure 2B (center panels). The right-most panels in Figure 2B represent average Z-scored gene expression profiles for the 5 patterns examined. Both the heatmaps and the data plots on the right side of Figure 2A reveal that Myr treatment enhances transcription at the 5 and 6 hour time points. This observation was tested by examining all genes found to be significant by the two-way ANOVA’s in the interaction term. For this test we plotted the log 2-fold changes of Myr treatment/vehicle treatment at each time point and color-coded the results if they were significantly down-regulated (blue) or up-regulated (orange) (*p* ≤ 0.05, pairwise Fisher’s LSD). As predicted, the predominant effect of Myr was to upregulate genes at the 5 and 6 h time points (Figure 2C). Functional overexpression analysis of significant DEGs at these two time points (called: I: 5 and 6(↑)) revealed a more generalized picture of the biological processes and cellular compartments responding to Myr (Figure 2C, right panel, all genes listed in Supplementary Table 2). As we discuss in detail below, up-regulation of genes starting between the 4th and 5th hour occurs when the majority of cells enter their second cell division cycle [7]. Thus, these data explain much of the effect of time and drug components within the interaction gene set.

We recently reported that Myr treatment has a notable effect on the size of most amino acid pools which remain significantly smaller in drug-treated cells starting before the 1 h time point, and remaining smaller over the six hour time course studied [7]. To determine if transcription has roles in lowering and maintaining smaller amino acid pools, we performed functional enrichment analysis on the 1570 genes in the Interaction group (Figure 2A) because this group captures a large fraction of genes responding differently over time with Myr treatment. We hypothesized that down-regulation of genes would imply a contribution to lower amino acid pools while up-regulation would imply the opposite effect or an attempt to restore pools to the size found in vehicle-treated cells. The KEGG pathway term ‘sce01100:Metabolic pathways’, containing 314 genes, is highly enriched in this gene set (*p* = 1,6E-27, FDR 1.54E-25) (Supplementary Table 2, INTERACTION group and 314 genes Metabolic pathways tabs). Importantly, there is strong enrichment for genes in other KEGG pathways including tryptophan, methionine and branched chain amino acid metabolism. Additionally, within the Interaction group the term ‘GO:0003333~amino acid transmembrane transport,’ with 16 genes, is enriched. A distinguishing feature of most genes in these three pathways plus the transporters is up-regulation in Myr-treated cells with many genes peaking at 5 and 6 h. These data suggest that Myr-treated cells are attempting to increase amino acid uptake and de novo synthesis as a way to increase amino acid pool levels, however, we consider other interpretations in the Discussion.

### Correlation analysis of transcriptomics data identifies clues to amino acid metabolism

**Figure 3:**
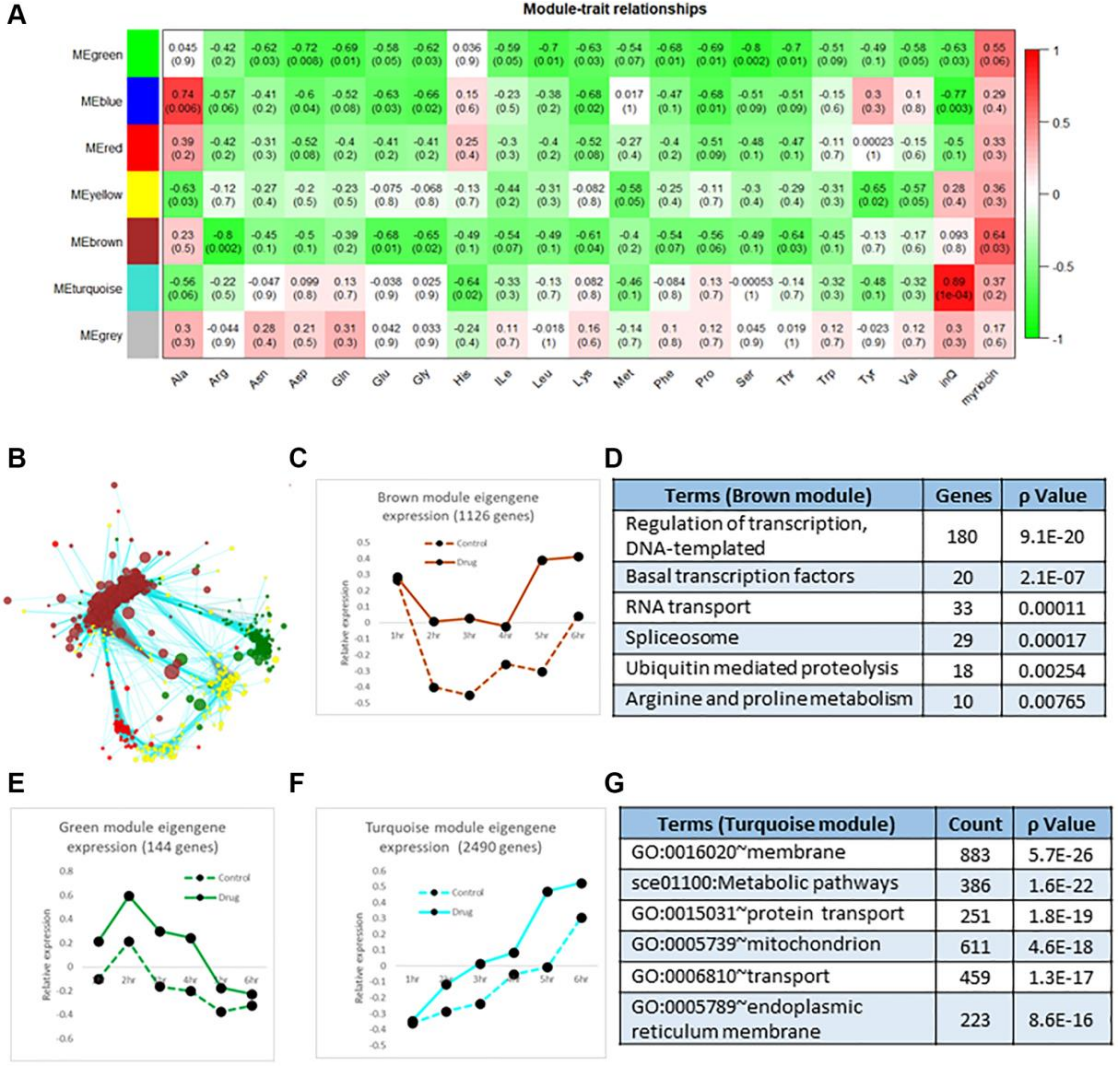
**Correlation analysis of transcriptomics data.** (**A**) The 7 color-coded modules (MEs, left side, Y-axis of heatmap), whose member genes are highly correlated over time, were analyzed for their correlation to each amino acid pool ((amino acids indicated on the X-axis, pool data are from [7]). The degree of correlation is indicated by the red-green (correlated-anticorrelated) scale at the right-side of the diagram and by numbers in columns which represent the correlation *p*-value and the correlation value (in parentheses). These values and the green highlighting indicate negative or anticorrelation: amino acid pools are small which transcripts are up-regulated, not down-regulated by Myr treatment. Additionally, the values in the column labeled 'InQ' represent the correlation between ME gene expression and the incubation time frame (1–6 hr) where the Turquoise module is the most correlated with time as shown graphically in panel F. The values in the column labeled 'myriocin' represent the association between ME gene expression and the absence/presence of myriocin where the no drug sample was set as 0 and the myriocin-treated sample was set as 1 for this calculation. This column indicates that the Green and Brown modules are the most anti-correlated (red shading) with amino acid pools, most of which are lowered by Myr treatment. (**B**) Network diagram showing the relationship of genes in the Green and Brown modules which are connected by genes in the Turquoise module. Genes are indicated by Nodes (circles) and relationships by edges. All genes and relationship values are presented in Supplementary Table 3. (**C**) Scatter plot of the Brown Eigengene across the 1–6 h time frame. (**D**) Enriched GO terms found in the Brown module. (**E**) Scatter plot of the Green Eigengene across the 1–6 h time. (**F**) Scatter plot of the Turquoise Eigengene across the 1–6 h time frame. (**G**) Enriched GO terms found in the Turquoise module. Genes used in calculating the mean Eigengene along with the 7 mean Eigengene values and their scatter plots are shown in Supplementary Table 3.

As another approach to understand effects of Myr on the transcriptome, we analyzed transcript data using the R program package of Weighted Gene Correlation Network Analysis, WGCNA [28], to identify clusters (modules) of highly correlated genes showing a similar response to Myr over the 1–6 h time course. A gene dendrogram produced by average linkage of hierarchical clustering revealed many modules with the seven most correlated having from 18 to 2490 genes (Supplementary Table 3, column L in Cluster Analysis tab, see also the Module Eigengenes tab). We then examined the relationship of these gene modules to free amino acid pools over the same 1–6 hr time course as an alternative way to determine if transcription played a role in lowering or maintaining smaller amino acid pools in drug-treated cells as we had previously found [7]. Results of this analysis are presented in Figure 3A as a heatmap plot. For amino acids (abbreviation shown on the X-axis), the numbers in a column represent the correlation *p*-value and the correlation (in parentheses) between a module eigengene (ME, Y-axis) expression value and an amino acid level. Here a ME corresponds to the first principal component of the expression matrix of the module. The ME can be considered the most representative gene in a module and, as used here, it represents the average effect of Myr on genes compared to the untreated drug control (Supplementary Table 3, Module Eigengenes tab). The heatmap shows that the Brown and Green modules with 1126 and 144 genes, respectively, have the greatest negative correlation to Myr treatment. That is, Myr maintains low pool levels but enhances the level of a transcript at one or more time points. The values in the ‘InQ’ column of the heatmap represent the correlation *p*-value and correlation value (parentheses) between the module eigengene expression and incubation time course (1–6 h). For these association calculations, the control (no drug) samples were set as 0 and Myr-treated samples were set as 1. Thus, the MEturquoise row has a strong correlation with time (Figure 3A, column inQ) as shown graphically in Figure 3F. The values in the 'myriocin' column represent the association between the module eigengene expression and the absence/presence of Myr and they indicate that the Green and Brown MEs are the most negatively correlated MEs.

As another way to represent the effects of Myr treatment on MEs and to detect interactions between MEs, we performed network analysis and visualized the results by using Cytoscape. One such network, representing the relationship between genes in the Brown and Green modules, includes the Turquoise module because genes interact with multiple genes in both the Brown and Green modules (Figure 3B). We further analyzed these three MEs by plotting the ME for each time point with and without Myr treatment (Figure 3C). For example, the ME representing the Brown module reveals Myr induces transcription starting at 4 h (Figure 3C) whereas there is no increase in amino acid pool size in drug-treated cells [7]. This opposite effect of Myr on transcription and amino acid pools corresponds to the negative correlation represented by the green shading in Figure 3A. The highest-ranking GO term in the Brown module is Regulation of Transcription (Figure 3D) where drug treatment enhances transcript levels particularly after 4 h, consistent with the ANOVA analysis (Figure 2B and 2C). A different negative correlation pattern is seen for the ME representing the small Green module where genes are up-regulated by Myr across the 1–6 h time frame compared to vehicle control but gene expression drops in both control and drug-treated samples starting at 2 h (Figure 3E). The main GO term in the Green module is Translation (Biological Process, *p* = 1.7E-88) along with Ribosome Assembly and related processes responding to drug-induced slowing of protein synthesis and growth rate [7]. Lastly, the large Turquoise module with 2490 genes captures Myr-induced transcriptional events involving processes or pathways or cell components each with more than 200 genes (Figure 3F, 3G). Interestingly, transcripts represented in the Turquoise module increase across the 1–6 h time frame with drug-treated cells always having higher transcript levels, substantiating the ANOVA analysis represented in Figure 1D (patterns T10a(↔), T&D04a(↑)., T&D05a(↑)), Figure 2B (patterns I05a(04a(↑)), I10a(04a(↑)) and I10a(10a(↑))) and Figure 2C. Functional enrichment analysis for all MEs in presented in Supplementary Table 3.

### Deubiquitination of Mup1 impairs Myr-enhanced longevity

**Figure 4:**
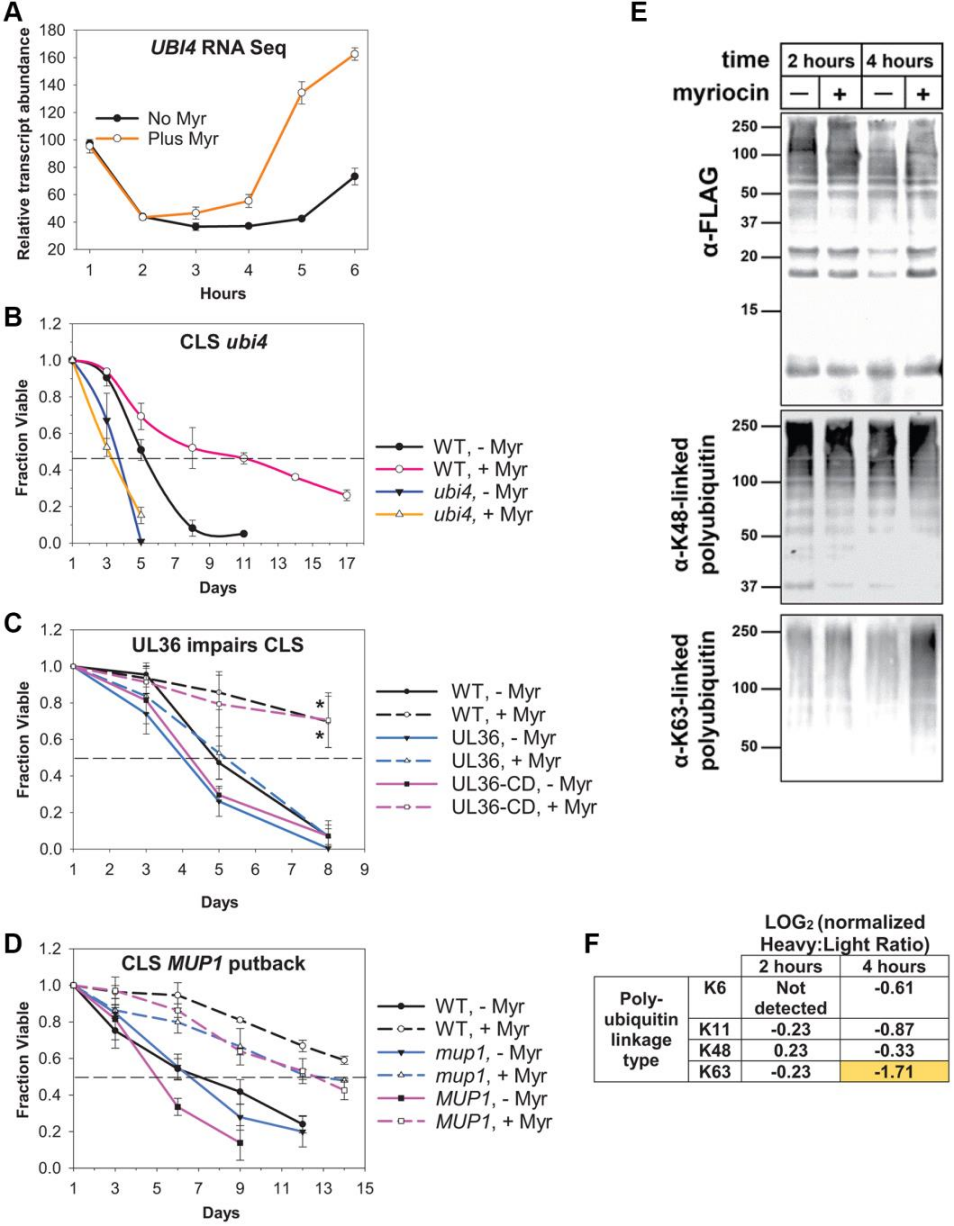
**Ubiquitin plays a central role in Myr-enhanced longevity.** (**A**) Summary of the relative abundance of *UBI4* transcripts across the 1–6 h time frame in Myr-treated or untreated cells. (**B**) CLS assay showing decreased survival of untreated (- Myr) *ubi4Δ* cells verses WT (BY4741) untreated cells and the complete lack of Myr-enhancement of lifespan in *ubi4Δ* cells compared to strong enhancement in WT cells. Error bars: SD (*N* = 3). (**C**) Data showing that removing ubiquitin from Mup1 impairs Myr-enhanced CLS. Cells having Mup1-pHlurion tagged with a UL36 deubiquitinase domain (DUB) are not able to respond to Myr treatment and enhance CLS. In contrast, replacing catalytically active UL36 with a catalytically dead UL36 domain (UL36-CD) restores Myr-enhanced lifespan. WT = BY4741 Mup1-pHluorin cells. Statistical significance at day 8 was determined (Student’s *t*-test) for Myr-treated WT vs. UL36 and UL36-CD vs. UL36 cells: *p*-values ≤ 0.024 and 0.032, respectively. Error bars: SEM (*N* = 3). (**D**) Lifespan assays reveal that deletion of *mup1* does not impair Myr-enhanced longevity. WT (BY4741) and *MUP1* putback (*mup1* allele replaced by *MUP1*) are control strains for the presence of a functional *MUP1* gens. Error bars: SEM (*N* = 3). (**E**) Affinity purified ubiquitin (from a yeast strain encoding N-terminally FLAG-tagged ubiquitin at two native, chromosomal ubiquitin genes) and blotted for total ubiquitin (top panel) captured as well as K63- and K48-linked polyubiquitin (middle and bottom panels, respectively) in cells treated on not treated with Myr after 2 or 4 h of cell growth. (**F**) SILAC-MS analysis of untreated (Heavy) or myriocin-treated (Light) yeast cells at the indicated time point. Heavy:Llight ratio quantifies relative abundance between the two samples. In this experiment, a negative LOG value indicates increased abundance in the myriocin-treated sample (and vice versa). All measurements were normalized to the Heavy:Light ratio for total ubiquitin (unmodified peptides), which did not significantly differ with Myr treatment at either 2 or 4 h post-myriocin treatment.

A drawback of WGCNA and pathway and pattern analyses is a reduced ability to identify significant biological features involving smaller numbers of genes or to identify genes that do not fit a specific pattern across the 1–6 h time-frame. To circumvent these limitations and to discover transcript changes with potential roles in lowering amino acid pools or novel roles in longevity enhancement, we examined genes with the highest possible significance (1E-16) in the Drug, Time and Interactions columns of data from the two-way ANOVA analysis of mRNA levels. This approach identified only 16 genes out of the 4964 genes (Supplementary Table 1, Filter tab, columns M, N and O). The *UBI4* gene, which is stress-induced and encodes for ubiquitin, captured our attention because stresses of various types have known roles in aging and longevity and we previously identified increased stress responses in Myr-treated cells [27, 29]. A defining feature of *UBI4* transcript abundance in our studies is a rapid increase at the 5–6 h time period (Figure 4A, statistical significance of Area Under Curve (AUC) 95% CI (difference of the means): −189.081 to −137.609, *p*-value = 0.00006). The path of the *UBI4* transcript level corresponds to the 5–6 h time period in which Myr has its most significant effect on transcription (Figure 2B and 2C) and it falls within Interaction pattern I: 5 and 6(↑) (Figure 2C and Supplementary Table 2, Webgestalt patterns combined tab). Proteolysis is a highly enriched GO term in this pattern of gene expression (Figure 2C, table on the right). The potential biological significance of the *UBI4* transcript pattern may relate to our previous analyses of Mup1-pHluorin trafficking. We found that the fluorescent signal decreases in the PM more rapidly during the 4–7 h time-frame in Myr-treated cells, in a dose-dependent manner, which requires ubiquitin conjugation for endocytosis to occur [7].

To determine if ubiquitination has roles in Myr-enhanced chronological lifespan (CLS), we examined untreated *ubi4Δ* cells and found a faster loss of viability compared to untreated BY4741 cells (Figure 4B, 50% survival day 3 vs. day 5, respectively). Additionally, the CLS of *ubi4Δ* cells treated with Myr does not increase and remains the same as untreated cells. Untreated wild-type (WT, BY4741) cells show 50% survival at day 5–6 which is extended to day 11 by Myr treatment (AUC, 95% CI: −6.828 to −2.840, *p*-value = 0.0025). From these data we conclude that *UBI4* is required for Myr to enhance CLS.

Since ubiquitin has many functions, we studied its role in ubiquitin-mediated endocytosis of Mup1 using the same strains as used in our published analyses of endocytosis [7]. Specifically, CLS was assayed using cells with chromosomal *MUP1* tagged with pHluorin-UL36 (catalytic active or catalytic dead, CD). UL36 is a viral deubiquitinase (DUB) that can reverse localized ubiquitin conjugation activity, thus protecting the fusion protein from ubiquitin-mediated degradation or trafficking events [30]. CLS is strongly enhanced by Myr treatment in two of these strains, namely WT cells (BY4741, *MUP1*-pHluorin) and cells with the catalytically dead UL36-CD domain (*MUP1*-pHluorin-UL36-CD) (Figure 4C, AUC of Myr-treated vs. untreated cells, 95% CL: −2.710 to −1.018, *p*-value = 0.0036 for WT and CI: −3.450 to −1.346, *p*-value = 0.0032 for UL36-CD). These significance values are probably an underestimate because both strains tend to regrow or gasp after day 8 which is why data are not shown beyond this time point. In contrast, CLS is only slightly enhanced by Myr treatment in cells with a catalytically active UL36 domain (Figure 4C, AUC, 95% CI: −1.864 to −0.0284, *p*-value = 0.05). Statistical significance was also evaluated by using a *t*-test for the day 8 survival values for Myr treated cells: WT vs. UL36 (*p*-value = 0.024) and UL36-CD vs. UL36 (*p*-value = 0.032). Taken together, these data show that Myr-enhanced longevity depends on Mup1 ubiquitination.

Our results reveal that Myr-enhanced longevity is prevented by Mup1 deubiquitination, which is a gain-of-function condition since it promotes PM stability. To determine how *MUP1* loss of function affects drug-enhanced longevity, we compared the CLS of *mup1Δ* cells to wild-type cells and cells with the *mup1Δ* allele replaced with wild-type *MUP1*. Myr treatment significantly enhanced CLS in each of the three strains (Figure 4D, for WT BY4741, *mup1Δ* and *MUP1* the AUC for Myr-treated vs. untreated cells, *p*-values ≤ 0.009, 0.026 and 0.0024, respectively). The viability curves for untreated *mup1Δ* and *MUP1* cells do not differ significantly (AUC, 95% CI: −1.682 to 0.860, *p*-value = 0.42). We conclude from these data that deleting *MUP1* does not prevent Myr-enhanced longevity.

Given the increased *UBI4* transcript level after 4–6 h of Myr treatment (Figure 4A), we hypothesized that Myr treatment might affect total ubiquitin and its cellular distribution. To test this, we affinity purified FLAG-ubiquitin (from yeast cells harboring N-terminal FLAG fusions at two endogenous ubiquitin-encoding loci (*RPS31* and *RPL40B*)) from untreated or Myr-treated yeast cells. Immunoblot analysis revealed that Myr treatment for 2 h or 4 h did not significantly alter the amount of FLAG-ubiquitin recovered or the amount of K48-linked ubiquitin polymers recovered (Figure 4E). In contrast, we observed a significant increase in K63-linked ubiquitin polymers after 4 h of Myr treatment (Figure 4E, right lane in bottom immunoblot), suggesting that increased conjugation of ubiquitin in K63-linked polymers is part of the cellular response to depletion of sphingolipids.

To confirm this result, and to resolve other linkage types, we performed *s*table *i*sotope *l*abeling with *a*mino acids in cell *c*ulture (SILAC) on yeast cells and subjected heavy-labelled and light-labelled cells to mock-treatment and Myr-treatment, respectively. At 2 hours and 4 hours of treatment, we collected cells, prepared lysates, and affinity purified FLAG-ubiquitin, followed by mixing, tryptic digestion, and processing of peptides for analysis by mass spectrometry. This SILAC-MS analysis resolved various peptides corresponding to the different linkage types of ubiquitin polymers, including K6-linked, K11-linked, K48-linked, and K63-linked polymers. Interestingly, we detected only modest changes in linkage types after 2 hours of Myr treatment, while 4 hours of Myr treatment resulted in increased formation of several polymer types, most notably for K63-linked ubiquitin polymers (Figure 4F). This finding is consistent with the immunoblot results shown in Figure 4E, confirming that yeast cells increase the formation of K63-linked ubiquitin polymers in response to Myr treatment. Taken together, these results suggest that yeast cells respond to Myr treatment by remodeling ubiquitin pools, partly by increasing production of ubiquitin via transcription of *UBI4* and partly by deploying existing ubiquitin pools to promote increased formation of K63-linked ubiquitin conjugates.

## DISCUSSION

We performed transcriptomics analysis to determine if Myr treatment caused a major transcriptional shift during a specific time-frame and to search for novel processes or pathways required for Myr-enhanced longevity. From our analysis, we are able to draw several conclusions. First, the major effect of Myr treatment is to up-regulate gene transcription (Figures 1D and 2B and 2C - heatmaps and graphs). Second, transcription is robustly up-regulated after 5 to 6 h of Myr treatment when the majority of cells advance through the second cell division cycle [7] (Figure 2B and 2C). Third, transcriptional changes are not the major statistically significant force promoting initial amino acid pool lowering since there is no enrichment for genes involved in amino acid metabolism in transcript patterns (Figure 1C and 1D). Additionally, enrichment analysis of genes in the Interaction group (Figure 2C) found enrichment for several amino acid metabolic pathways and transmembrane transporters (Supplementary Table 2, Tabs labelled INTERACTION group and 314 Metabolic pathways – see yellow highlighting), but these genes are mostly up-regulated by Myr treatment, suggesting that cells are attempting to increase amino acid pool size. Furthermore, correlation analysis found an anticorrelation or negative relationship between gene modules and amino acid pool sizes (Figure 3A). For example, the large Turquoise module containing genes up-regulated across the 1–6 h time-frame (Figure 3F) reveals enrichment for metabolic pathways including ones for amino acid metabolism (Figure 3G and Supplementary Table 3, tab labelled GO terms Turquoise module). Notably, we showed previously [7] that amino acid pool lowering, starting after about 2 h of cell growth, is at least partly due to inactivation of amino acid transporters at the PM following Myr treatment. Fourth, ubiquitination of the methionine transporter Mup1 at the PM is vital for Myr-enhanced longevity (Figure 4B, 4C). However, we cannot altogether exclude a role for transcription of a small number of genes as playing roles in reducing or maintaining smaller amino acid pools. Lastly, Myr treatment strongly up-regulates K63-linked ubiquitination of proteins (Figure 4E, 4F). Importantly, we have also recently reported that reducing sphingolipid synthesis inhibits the endocytosis of many nutrient transporters, while specifically promoting endocytic clearance of the methionine transporter Mup1 [31]. Taken together, our analysis reveals a complex cellular response to Myr that involves modulation of several biological processes including endocytic trafficking, proteostasis, and amino acid homeostasis.

The transcriptomics data presented here provide a valuable resource not only for constructing a more mechanistic understanding of how Myr treatment reprograms yeast cells to live longer, but it also provides a strategy for discovering new mechanisms to promote longevity. For instance, GO terms involving transcription are highly enriched in the D(↑) temporal pattern (Table 1, genes in this pattern are listed in Supplementary Table 2) where they represent 4 of the 6 top GO terms. Many of these encode transcription regulators including *GCN4* whose transcription is up-regulated by amino acid starvation in order to promote de novo amino acid biosynthesis [32]. Thus, the failure of cells to increase amino acid pools in response to increased *GCN4* transcription implies activation of non-transcriptional mechanisms by Myr treatment to lower and maintain smaller amino acid pools. The GO term Metabolic Pathways (314 genes) was enriched in the Interaction group during a part or all of the 1–6 h time-frame (Figure 2C, Supplementary Table 2, INTERACTION group and 314 genes Metabolic pathways tabs). Functional enrichment analysis of these 314 genes identified KEGG pathways for carbon metabolism, secondary metabolites, amino acid metabolism (tryptophan, methionine, branched-chain amino acids, lysine, arginine, proline, histidine) and other types of metabolism as being significantly enriched. Several of these pathways have known roles in longevity (e.g., methionine, branched-chain amino acids, glycogen, trehalose metabolism, etc.), suggesting that the Myr-sensitive pathways defined in this work are novel but include elements of pathways defined in prior work as having roles in longevity. Endocytosis is another GO term found to be highly enriched in the Interaction analysis of transcripts across the 1–6 h time-frame (Supplementary Table 2, INTERACTION group tab). GO term analysis of this group of 54 genes found enrichment for genes involved in ubiquitin-mediated endocytosis which provide clues to identify the proteins, lipids and cellular machinery controlling the Myr-induced endocytosis of Mup1 that we previously observed [7]. Detailed analysis of the gene interaction network shown in Figure 3B should aid in identifying the most important Node/Hub genes along with their edges potentially involved in controlling Mup1 endocytosis. Lastly, a novel feature of the Interaction group is enrichment of GO terms representing nearly every cellular component, many of which are membrane-bound (Supplementary Table 2, INTERACTION group tab). We hypothesize that Myr treatment starts early (~ 5 h or the second cell division) to widely and coordinately rewire cellular physiology towards enhanced longevity well before many hallmarks of aging and longevity are detectible. For instance, we previously showed that autophagy begins after 3–5 cell divisions [27] whereas many autophagy genes in the INTERACTION group are strongly up-regulated in the second cell division (4–5 h) following Myr treatment. Our transcriptomics dataset will be a valuable resource for determining if coordinated rewiring is promoted by cell cycle mechanisms or cross-talk between cellular components or by both mechanisms.

In addition to the limitations inherent in WGCNA, pattern and pathway analyses such as genes not falling into any category, there are other potential limitations in our analyses to consider. For example, improved detection and resolution of drug effects might be obtained with increased cell cycle synchronization, more time points and more sample replicates but these are not likely to alter the overall results of this work. Additionally, analysis of transcripts before 1 hr might identify potential mechanisms for maintaining smaller amino acid pools in drug-treated cells. Adding Myr after cells have reentered the cell division cycle would be an alternative approach for studying early effects of Myr on transcription, but integrating such data with results presented here and in our earlier studies could be difficult [7, 27, 29].

Healthy proteostasis relies on proper functioning of protein degradation networks to maintain proteome quality control and prevent accumulation of misfolded and damaged proteins. Thus, a decline in proteostasis is a hallmark of aging and age-related diseases [33]. Analysis of the yeast transcriptional response to Myr treatment revealed a significant induction of the mRNA transcript encoded by *UBI4* (Figure 4A) – one of four genes in the yeast genome encoding for ubiquitin. The other three [34] ubiquitin genes (*RPS31*, *RPL40A*, and *RPL40B*) encode ubiquitin fusions to ribosomal subunits and generate most cellular ubiquitin in normal growth conditions, which inherently couples the translational and degradative capacities in the cell. By comparison, *UBI4* encodes a linear (heat-to-tail) ubiquitin pentamer that does not contribute significantly to the total pool of ubiquitin in normal growth conditions. However, in conditions of heat stress, oxidative stress, or starvation *UBI4* is transcriptionally induced which serves to increase the degradation capacity of the cell and uncouple it from ribosome biogenesis and translational capacity [34, 35]. Thus, our observation that Myr induces transcription of *UBI4* but not the other genes encoding ubiquitin (transcript levels of other ubiquitin genes can be displayed by using the ‘Graph Reporter’ tab in Supplementary Table 1) is suggestive of a proteotoxic and/or starvation stress response involving activation of protein degradation networks and sizeable proteome remodeling. The importance of *UBI4* in Myr-enhanced longevity is underscored by our finding that the CLS of untreated *ubi4Δ* yeast cells decreased compared to WT cells and was not significantly enhanced by Myr treatment (Figure 4B). In addition, our finding that cells with chromosomal *MUP1* tagged with pHluorin-UL36 fail to show Myr-enhanced CLS (Figure 4C) suggests a novel role for ubiquitination in longevity. Importantly, we previously reported that UL36 fusion to Mup1 impedes its Myr-induced endocytosis [7], underscoring the linkage of ubiquitin-mediated endocytosis of Mup1 to Myr-mediated longevity. One limitation of this analysis is the potential for proximal effects on other proteins in the vicinity of the Mup1-pHluorin-UL36 protein [30, 36]. Thus, we cannot exclude the possibility that ubiquitination of Mup1-associated factors is critical for Myr-mediated longevity. Future studies will be required to evaluate if such proximal effects are involved in the impairment of Myr-enhanced lifespan. The outcomes of these studies will potentially provide new targets or strategies for improving human healthspan.

## MATERIALS AND METHODS

### Strains, culture conditions, lifespan assays and statistical significance

Strains (Table 3), culture conditions, lifespan assays and their statistical significance were similar to ones described previously [7]. Specifically, yeast cells, made prototrophic by transformation with pHLUM (carries *HIS3*, *LEU2*, *URA3* and *MET*, Addgene, Watertown, MA, USA [37]) and stored at −80°C, were streaked onto minimal glucose plates and incubated at 30°C for 3 days. One to three colonies were inoculated into 5 ml of SD complete medium (SDC) [38] and incubated on a gyratory water bath shaker for 18–24 h at 30°C. Typical culture densities were 7–9 A600 nm/ml (1.5 × 10^7^ cells/A600 nm). Cells were diluted into 25 ml fresh SDC warmed to 30°C in a 125 ml long-neck flask, containing vehicle (EtOH) or Myr (details described in the next paragraph), to give a starting cell density of 0.15 A600 nm. Flasks were incubated at 30°C on a gyratory air-bath shaker (200 rpm). After 72 h of incubation cells were diluted (1:50 × 1:100) and from 10 to 40 μL was spread on a YPD plate [38] and incubated 48–72 h at 30°C followed by colony counting. CLS assays were performed at least twice using triplicate cultures. Concentrations of Myr used in CLS assays ranged from 475–525 ng/ml depending on the sensitive of strains compared to wild-type, prototrophic BY4741. Drug-sensitivity was measured by culture density (A600 nm) after 24 h of growth using cultures started at 0.15 A600 nm units of cells/ml. All BY4741 yeast strains used for lifespan assays and for RNA extraction were made prototrophic by transformation with the pHLUM plasmid. The DUB (UL36) fusion yeast strains used in this study were generated by homologous recombination in BY4741 and SEY6210 background strains using the reagents and strategy previously described (Hepowit et al. 2022). The *MUP1* knock-in strain (NHY945) was generated in BY4741 by swapping the endogenous *MUP1* coding region with NATMX (NHY930), shuffling the chromosomally integrated *NATMX* with *URA3* (NHY938.1), and snipping out *URA3* by homologous reintegration of a PCR-amplified *MUP1* coupled with counter selection on 5-fluoroorotic acid (5-FOA) synthetic media plate. A two-tailed Student’s *t*-test and Area Under the Curve (AUC) (Sigma Plot) were used to evaluate statistical significance of CLS assays done with at least 3 biological replicates. Results were verified by one or more repeat experiments.

**Table 3:** Strains used in this study.

**Strain**	**Genotype**	**References**
BY4741	*MATa his3-Δ1 leu2-Δ0 ura3-Δ0 met15-Δ0*	[43]
SEY6210	*MATalpha leu2-2, 112 ura3-52 his 3delta200 trp1-delta901 lys2-801 suc2-delta9*	[44]
NHY413	SEY6210 *Mup1-pHluorin::NATMX*	[7]
NHY414	SEY6210 *Mup1-pHluorin::NATMX Vph1-MARS::TRP1*	[7]
NHY447	SEY6210 *Mup1-pHluorin-UL36(N-term 15-260 HSV1 UL36, active)::KANMX*	[7]
NHY431	SEY6210 *Mup1-pHluorin-UL36(N-term 15-260 HSV1 UL36 C40S, inactive)::KANMX*	[7]
RCD2073	BY4741 *MATa his3-Δ1 leu2-Δ0 ura3-Δ0 met15-Δ0 ubi4Δ::KAN*	[45]
NHY415	BY4741 *MATa his3-Δ1 leu2-Δ0 ura3-Δ0 met15-Δ0 Mup1-pHluorin (NATMX)*	This study
NHY425	BY4741 *MATa his3-Δ1 leu2-Δ0 ura3-Δ0 met15-Δ0 Mup1-pHluorin-UL36 (KANMX)*	This study
NHY426	BY4741 *MATa his3-Δ1 leu2-Δ0 ura3-Δ0 met15-Δ0 Mup1-pHluorin-UL36 catalytic dead (KANMX)*	This Study
NHY930	BY4741 *MATa his3-Δ1 leu2-Δ0 ura3-Δ0 met15-Δ0 Δmup1::NATMX*	This Study
NHY938.1	BY4741 *MATa his3-Δ1 leu2-Δ0 ura3-Δ0 met15-Δ0 Δmup1::URA3*	This Study
NHY938.2	BY4741 *MATa his3-Δ1 leu2-Δ0 ura3-Δ0 met15-Δ0 Δmup1::URA3*	This Study
NHY945	BY4741 *MATa his3-Δ1 leu2-Δ0 ura3-Δ0 MUP1 knockin*	This Study
JMY1312	SEY6210 *MATα arg4::KANMX FLAG-RPS3::TRP1, FLAG-RPL40B::TRP1*	[42]

Budding yeast mRNA-enriched profiles were generated from 42 individual samples of prototrophic BY4741 cells, including 3 replicates at 7 time points and 2 treatments (control and drug-treated). Culture conditions were similar to ones described previously (Hepowit et al. 2021, Aging) except for the following modifications. Prototrophic BY4741 yeast cells were grown in 200 ml of SDC culture medium in a 1 L flask with the medium heated to 30°C before addition of EtOH (final concentration of 0.3% for control samples). For drug-treated samples, myriocin was added after addition of EtOH to give final concentrations of 0.3% EtOH and 700 ng/ml myriocin. Lastly, cells from a saturated overnight culture were diluted into the 200 ml cultures to give an initial A600 nm units/ml of 0.15. Flasks were incubated at 30°C and 200 rpm on a rotary shaker. Control and myriocin-treated cells were harvested at time 0, 1, 2, 3, 4, 5 and 6 h (all time points for each replication were from the same culture flask).

### RNA extraction

Culture conditions, medium and prototrophic BY4741 cells were the same as for lifespan and amino acid pool assays [7]. RNA was extracted from 5 A600 nm unit/ml of yeast cells harvested by filtration on a membrane filter at each time point (1–6 h, Figure 1A) [7]. Filtered cells were washed once with 5 ml of ice-cold nanopure water and the filter was quickly transferred to a chilled 1.5 ml microfuge tube containing 0.5 ml cold nanopure water. Tubes were vortexed 10 sec followed by centrifugation for 15 sec. Supernatant fluid (450 μl) was transferred to a new tube and frozen in a dry-ice EtOH bath followed by storage at −80°C. Cold acid-washed glass beads (300 μl, 0.5 mm dia.) were added to a frozen cell pellet followed by addition of 300 μl of RLT buffer (RNAeasy mini kit, Qiagen, Germantown, MD, USA). Tubes were vortexed 5 min at room temperature and placed on ice for 1 min. After 4 cycles 300 μl of ice-cold RLT buffer was added followed by mixing and centrifugation at 13,000 × g for 2 min at room temperature. Supernatant fluid (450 μl) was transferred to a new microfuge tube and then mixed with 1 ml of 70% (v/v) EtOH before transfer to a RNeasy spin column and processed according to the manufacturer’s instructions. Processed samples were frozen in a dry-ice EtOH bath and stored at −80°C.

### RNA-seq analysis

RNA sequencing was performed on total RNA samples at the Roy J. Carver Biotechnology Center at the University of Illinois. Two different mixes of ERRC spike-in RNA controls were added to the samples; one mix for the control samples, and one mix for the drug-treated samples. Libraries were constructed with Illumina’s ‘TruSeq Stranded mRNAseq Sample Prep kit’. Each library was quantitated by qPCR and sequenced on one lane for 151 cycles from each end of the fragments on a HiSeq 4000 using a HiSeq 4000 sequencing kit version 1. Fastq files were generated and demultiplexed with the bcl2fastq v2.17.1.14 Conversion Software (Illumina). Each sample’s pair of fastq files were run through trimmomatic 0.36 to first remove any remaining standard Illumina PE v3 adapters, then trim bases from both ends with quality scores below 28, and finally to remove individual reads shorter than 30 bp and their paired read, regardless of length (parameters ILLUMINACLIP:/home/apps/trimmomatic/trimmomatic-0.36/adapters/TruSeq3-PE.fa:2:15:10 TRAILING:28 LEADING:28 MINLEN:30). Paired reads per sample were pseudo-aligned to the Yeast R64 transcriptome and 92 ERRC spike-in control sequences using Salmon 0.8.2 with parameters -l A –numbootstraps = 30 --seqBias –gcBias.

Resulting FASTQ files mapped to the yeast genome (R64), resulted in count files and normalized using the transcripts per million (TPM) algorithm [39] using WebMev [40]. Resulting data were downloaded as flat files and loaded into Excel for further analysis. From a total of 6198 mapped genes, 5169 were uniquely annotated with gene symbols and had sufficient non-zero readings for further analysis. The filtered data were analyzed by two-way ANOVA for the main effects of drug and time, as well as for interaction and assigned a *p*-value. A gene found to be significant in any of these three categories was referred to as differentially expressed (DEG). Significant by the time term or both the drug and time term, data were further analyzed by post-hoc pairwise Fisher’s protected Least Significant Difference (pLSD), and log 2-fold change comparison to further isolate the effects of drug over time.

To identify DEGs that change up or down over time we built a ‘template assignment tool’ that took each DEG and attempted to assign it to each of 63 patterns. The pattern to which a DEG fit best (by Pearson’s correlation, had to be R > 0.85) became the pattern to which it was assigned. This is reported in Figure 1C (99.86% of all DEGs were assigned to a template). To determine which templates had significantly more DEGs than expected by chance, we ran a binomial test- this is the basis for some templates being bolded and having an asterisk in Figure 1C- they had a binomial *p* < 0.01. The 12 patterns of expression that had more DEGs than expected by chance assigned to them explained the majority (77%) of all DEGs. Data have been deposited in the GEO (GSE199904) [NCBI tracking system #22817261]. GO terms were analyzed by using DAVID software [41] or online WebGestalt.

### Gene regulatory network using WGCNA

The WGCNA (v1.70-3) [28] was used to identify gene modules and build unsigned co-expression networks, which include negative and positive correlations. Briefly, WGCNA constructs a gene co-expression matrix, uses hierarchical clustering in combination with the Pearson correlation coefficient to cluster genes into groups of closely co-expressed genes termed modules, and then uses singular value decomposition (SVD) values as module eigengenes to determine the similarity between gene modules or to calculate association with sample traits (for example, incubation time or amino acid levels). The top 2,000 variable genes were used to identify gene modules and network construction. Soft power 8 was chosen by the WGCNA function pick SoftThreshold. Then, the function TOMsimilarityFromExpr was used to calculate the TOM similarity matrix via setting power = 8, networkType = "signed. The distance matrix was generated by subtracting the values from similarity adjacency matrix by one. The function flashClust (v.1.01) was used to cluster genes based on the distance matrix, and the function cutreeDynamic was utilized to identify gene modules by setting deepSplit = 3. Cytoscape (v.3.8.2) was applied for the network visualizations.

### Analysis of ubiquitin and its linkage types

For immunoblotting assays 5 A600 nm unit of cells were precipitated in 10% trichloroacetic acid on ice for 30 min. The protein precipitate was washed twice with ice-chilled acetone, lyophilized by vacuum centrifugation, and resuspended in urea sample buffer (75 mM Tris-HCl [pH 6.8], 6 M urea, 1 mM EDTA, 3% SDS, 20% glycerol, bromophenol blue), heated at 65°C for 5 min, and vortexed for 5 min. Proteins were separated by SDS-PAGE and transferred onto Immobilon-P^SQ^ membrane (0.2 μm; Millipore). Immunoblotting was performed using the following primary antibodies: anti-ubiquitin (1:10,000; LifeSensors; MAb; clone VU-1), anti-K48 (1:10,000; Cell Signaling; RAb; clone D9D5), and anti-K63 (1:4000; EMD Millipore; RAb; clone apu3). Secondary antibodies were anti-mouse (IRDye 680RD-Goat anti-mouse) or anti-rabbit (IRDye 800CW-Goat anti-rabbit) obtained from LI-COR Biosciences). Blots were scanned using Odyssey CLx and signal fluorescence was visualized using Image Studio Lite (LI-COR Biosciences). Ubiquitin linkage types in JMY1312 yeast cells were determined and quantified by SILAC-based mass spectrometry as previously described except for a change in the cell lysis buffer: 50 mM Tris-HCl [pH7.5], 150 mM NaCl, sodium pyrophosphate, 20 mM β-glycerophosphate, 2 mM sodium orthovanadate, 1 mM phenylmethylsulfonyl fluoride, 0.2% NP-40, 10 mM iodoacetamide, 20 μM MG132, 1 mM 1,10-phenanthroline, 1× EDTA-free protease inhibitor cocktail (Roche), 1× PhosStop (Roche) [42].

## Supplementary Materials

Supplementary Table 1

Supplementary Table 2

Supplementary Table 3
